# P-2195. Influence of Nucleos(t)ide Analogue Use with Bulevirtide on Treatment Outcomes in Chronic Hepatitis Delta

**DOI:** 10.1093/ofid/ofaf695.2358

**Published:** 2026-01-11

**Authors:** Pietro Lampertico, Maurizia Brunetto, Maria Buti, Soo Aleman, Pavel Bogomolov, Vladimir Chulanov, Nina Mamonova, Viacheslav Morozov, Olga Sagalova, Tatiana Stepanova, Renee-Claude Mercier, Mingyang Li, Amos Lichtman, Dmitry Manuilov, Heiner Wedemeyer, Tarik Asselah, Fabien Zoulim

**Affiliations:** CRC “A. M. and A. Migliavacca” Center for Liver Disease, Department of Pathophysiology and Transplantation, University of Milan, Milan, Lombardia, Italy; Hepatology Unit, Reference Center of the Tuscany Region for Chronic Liver Disease and Cancer, University Hospital of Pisa, Pisa, Italy; Department of Clinical and Experimental Medicine, University of Pisa, Pisa, Italy, Pisa, Toscana, Italy; Hospital Universitario Vall d’Hebron, Barcelona, Spain; CIBEREHD del Instituto Carlos III, Madrid, Spain, Barcelona, Catalonia, Spain; Department of Infectious Diseases, Karolinska University Hospital/Karolinska Institutet, Stockholm, Sweden, Stockholm, Sodermanlands Lan, Sweden; M.F. Vladimirsky Moscow Regional Research and Clinical Institute, Moscow, Russian Federation, Moscow, Moskva, Russia; Sechenov University, Moscow, Moskva, Russia; FSBI National Research Medical Center for Phthisiopulmonology and Infectious Diseases of the Ministry of Health of the Russian Federation, Moscow, Russian Federation, Moscow, Moskva, Russia; LLC Medical Company Hepatolog, Samara, Russian Federation, Samara, Samara, Russia; South Ural State Medical University, Chelyabinsk, Russian Federation, Chelyabinsk, Moskva, Russia; LLC Clinic of Modern Medicine, Moscow, Russian Federation, Moscow, Moskva, Russia; Gilead Sciences, Inc., Foster City, CA; Gilead Sciences, Inc., Foster City, CA; Gilead Sciences, Inc., Foster City, CA; Gilead Sciences, Inc., Foster City, CA; Clinic for Gastroenterology, Hepatology, Infectious Diseases, and Endocrinology, Hannover Medical School, Hannover, Germany, Hannover, Sachsen, Germany; Université de Paris-Cité. Hôpital Beaujon, Paris, France., Not Applicable, France; Hospital Croix Rousse, Lyon, Rhone-Alpes, France

## Abstract

**Background:**

Hepatitis delta virus (HDV) causes the most severe form of viral hepatitis. Although nucleos(t)ide analogues (NAs) are the first-line treatment for chronic hepatitis B virus (HBV) infection, NAs have not been effective in reducing HDV RNA levels in patients with chronic hepatitis delta (CHD). Recent HBV/HDV treatment guidelines recommend NAs for patients with CHD with or without cirrhosis but with HBV DNA levels ≥2000 IU/mL. We evaluated the virologic outcomes of bulevirtide (BLV) treatment over a 96-week period, comparing its effects when administered with and without NAs.

**Table.**
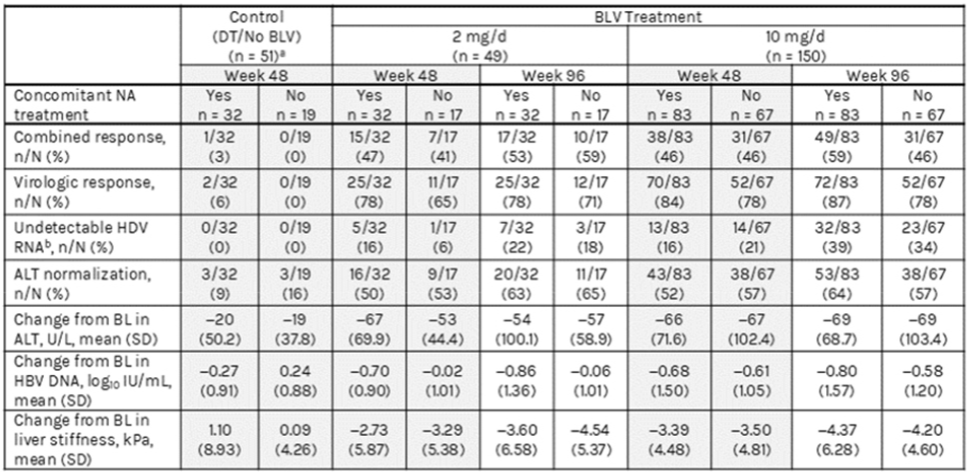
Treatment Outcomes at Week 48 for All Groups and Week 96 (BLV 2 and 10 mg) by Concomitant NA Treatment (Pooled Analysis of Studies MYR204 and MYR301) aThe DT group included 51 patients who did not receive BLV for 48 weeks; 50 of these patients received BLV 10 mg from week 48 to 144 and are included in the 10mg/d group. Change from baseline for these 50 patients was reset at week 48 in the BLV 10 mg/d group. bDefined as ALT, alanine aminotransferase; BL, baseline; BLV, bulevirtide; d, day; DT, delayed treatment; HBV, hepatitis B virus; HDV, hepatitis delta virus; NA, nucleos(t)ide analogue.

**Methods:**

Data from 2 randomized studies (MYR204 [NCT03852433]; MYR301 [NCT03852719]) were pooled for patients receiving BLV monotherapy 2 or 10 mg/day (d) for 96 weeks, including a delayed treatment (DT) arm with no BLV treatment for 48 weeks as a control. NA treatment was allowed per treatment guidelines. Treatment outcomes included undetectable HDV RNA < the lower limit of quantitation (50 IU/mL, target not detected), virologic response (VR; undetectable HDV RNA or ≥2 log_10_ IU/mL decline from baseline [BL]), alanine aminotransferase (ALT) normalization, change from BL in ALT, combined response (VR and ALT normalization), and change from BL in HBV DNA and liver stiffness, all assessed over 96 weeks with vs without NAs.

**Results:**

We included patients in 3 groups: BLV 2 mg/d (n = 49), BLV 10 mg/d (n = 150), and the DT arm (n = 51). Overall, NAs were used in 32/49 (65%), 83/150 (55%), and 32/51 (63%) patients in the BLV 2 mg, 10 mg, and DT groups, respectively. BL characteristics were comparable, except the proportions of patients with cirrhosis, prior interferon use, and mean liver stiffness were higher in those with NAs (54%; 61%; 14.8 kPa) vs without NAs (30%; 41%; 13.4 kPa). The most common therapy was tenofovir based (97/114 [85%]). Treatment outcomes with vs without NAs at week 48 for the DT group and week 96 for the BLV 2 and 10 mg groups are shown in the table.

**Conclusion:**

BLV given for 96 weeks is effective in achieving HDV virologic outcomes and ALT normalization in patients with CHD regardless of NA use. While the addition of NA therapy did not substantially improve HDV outcomes, it did contribute to a greater reduction in HBV DNA levels.

**Disclosures:**

Pietro Lampertico, MD, AbbVie: Speaking/teaching fees/advisory committee/review panel|Aligos Therapeutics: Speaking/teaching fees/advisory committee/review panel|Alnylam Pharmaceuticals: Speaking/teaching fees/advisory committee/review panel|Antios Therapeutics: Speaking/teaching fees/advisory committee/review panel|Arrowhead Pharmaceuticals: Speaking/teaching fees/advisory committee/review panel|Bristol Myers Squibb: Speaking/teaching fees/advisory committee/review panel|Eiger Biopharmaceuticals: Speaking/teaching fees/advisory committee/review panel|Gilead Sciences, Inc.: Speaking/teaching fees/advisory committee/review panel|GSK: Speaking/teaching fees/advisory committee/review panel|Janssen: Speaking/teaching fees/advisory committee/review panel|Merck Sharp & Dohme: Speaking/teaching fees/advisory committee/review panel|MYR GmbH: Speaking/teaching fees/advisory committee/review panel|Roche: Speaking/teaching fees/advisory committee/review panel|Spring Bank Pharmaceuticals: Speaking/teaching fees/advisory committee/review panel Maurizia Brunetto, MD, AbbVie: Consulting/speaker bureaus|Eisai–Merck Sharp & Dohme: Consulting/speaker bureaus|Gilead Sciences, Inc.: Consulting/speaker bureaus|Janssen: Consulting/speaker bureaus|Roche: Consulting/speaker bureaus Maria Buti, MD, PhD, AbbVie: Speaker fees/research support/consulting fees|Gilead Sciences, Inc.: Speaker fees/research support/consulting fees|Janssen: Speaker fees/research support/consulting fees Soo Aleman, MD, PhD, AbbVie: Grant/Research Support|AbbVie: Honoraria|Biogen: Honoraria|Gilead Sciences, Inc.: Grant/Research Support|Gilead Sciences, Inc.: Honoraria|Merck Sharp & Dohme: Honoraria Vladimir Chulanov, MD, PhD, AbbVie: Consultant/sponsored lectures|AstraZeneca: Consultant/sponsored lectures|Bristol Myers Squibb: Consultant/sponsored lectures|Gilead Sciences, Inc.: Consultant/sponsored lectures|GSK: Consultant/sponsored lectures|Hepatera: Consultant/sponsored lectures|Merck Sharp & Dohme: Consultant/sponsored lectures|Roche: Consultant/sponsored lectures|R-Pharm: Consultant/sponsored lectures Renee-Claude Mercier, PharmD, Gilead Sciences, Inc.: Employee|Gilead Sciences, Inc.: Stocks/Bonds (Public Company) Mingyang Li, PhD, Gilead Sciences, Inc.: Employee|Gilead Sciences, Inc.: Stocks/Bonds (Public Company) Amos Lichtman, MPH, MD, Gilead Sciences, Inc.: Employee|Gilead Sciences, Inc.: Stocks/Bonds (Public Company) Dmitry Manuilov, MD, Gilead Sciences, Inc.: Employee|Gilead Sciences, Inc.: Stocks/Bonds (Public Company) Heiner Wedemeyer, MD, Abbott: Advisor/Consultant|Abbott: Honoraria|AbbVie: Advisor/Consultant|AbbVie: Honoraria|Aligos Therapeutics: Advisor/Consultant|Arbutus Biopharma: Advisor/Consultant|Boehringer Ingelheim: Advisor/Consultant|Boehringer Ingelheim: Honoraria|Bristol Myers Squibb: Advisor/Consultant|Bristol Myers Squibb: Honoraria|Dicerna Pharmaceuticals: Advisor/Consultant|Gilead Sciences, Inc.: Advisor/Consultant|Gilead Sciences, Inc.: Honoraria|Johnson & Johnson/Janssen-Cilag: Advisor/Consultant|Johnson & Johnson/Janssen-Cilag: Honoraria|Merck/Schering-Plough: Advisor/Consultant|Merck/Schering-Plough: Honoraria|MYR GmbH: Advisor/Consultant|MYR GmbH: Honoraria|Novartis: Advisor/Consultant|Novartis: Honoraria|Roche: Advisor/Consultant|Roche: Honoraria|Siemens: Advisor/Consultant|Siemens: Honoraria|Transgene: Advisor/Consultant|Transgene: Honoraria|ViiV Healthcare: Advisor/Consultant|ViiV Healthcare: Honoraria|Vir Biotechnology: Advisor/Consultant Tarik Asselah, MD PhD, AbbVie: Speaker/investigator|Eiger Biopharmaceutical: Speaker/investigator|Gilead Sciences, Inc.: Speaker/investigator|Janssen: Speaker/investigator|Merck: Speaker/investigator|Myr Pharmaceutical: Speaker/investigator|Roche: Speaker/investigator Fabien Zoulim, MD, PhD, Aligos Therapeutics: Advisor/Consultant|Aligos Therapeutics: Grant/Research Support|Assembly Biosciences: Advisor/Consultant|Assembly Biosciences: Grant/Research Support|Gilead Sciences, Inc.: Advisor/Consultant|GSK: Advisor/Consultant|Vir Biotechnology: Advisor/Consultant

